# Integrative Analysis of Differentially Expressed Genes in Time-Course Multi-Omics Data with MINT-DE

**DOI:** 10.21203/rs.3.rs-3806701/v1

**Published:** 2024-01-01

**Authors:** Hao Xue, Sofie Y. N. Delbare, Martin T. Wells, Sumanta Basu, Andrew G. Clark

**Affiliations:** 1 Department of Computational Biology, Cornell University, 526 Campus Rd, Ithaca, 14853, NY, USA.; 2 Department of Statistics and Data Science, Cornell University, 129 Garden Ave, Itahca, 14853, NY, USA.

**Keywords:** Multi-omics, Edgington’s method, Time-course, Translational regulation

## Abstract

**Background:**

Time-course multi-omics experiments have been highly informative for obtaining a comprehensive understanding of the dynamic relationships between molecules in a biological process, especially if the different profiles are obtained from the same samples. A fundamental step in analyzing time-course multi-omics data involves selecting a short list of genes or gene regions (“sites”) that warrant further study. Two important criteria for site selection are the magnitude of change and the temporal dynamic consistency. However, existing methods only consider one of these criteria, while neglecting the other.

**Results:**

In our study, we propose a framework called MINT-DE (**M**ulti-omics **IN**tegration of **T**ime-course for **D**iffierential **E**xpression analysis) to address this limitation. MINT-DE is capable of selecting sites based on summarized measures of both aforementioned aspects. We calculate evidence measures assessing the extent of differential expression for each assay and for the dynamical similarity across assays. Then based on the summary of the evidence assessment measures, sites are ranked. To evaluate the performance of MINT-DE, we apply it to analyze a time-course multi-omics dataset of *Drosophila* development. We compare the selection obtained from MINT-DE with those obtained from other existing methods. The analysis reveal that MINT-DE is able to identify differentially expressed time-course pairs with the highest correlations. Their corresponding genes are significantly enriched for known biological functions, as measured by gene-gene interaction networks and the Gene Ontology enrichment.

**Conclusions:**

These findings suggest the effectiveness of MINT-DE in selecting sites that are both differentially expressed within at least one assay and temporally related across assays. This highlights the potential of MINT-DE to identify biologically important sites for downstream analysis and provide a complementarity of sites that is neglected by existing methods.

## 1 Introduction

Multi-omics assays have facilitated a comprehensive analysis of biological systems and have revealed additional biological insights [1, 2]. This is a key step towards gaining a global understanding of the underlying biological mechanisms, because integrating different omics datasets provides useful non-redundant readouts which cannot be obtained from any single modality alone. However, some biological mechanisms entail complex dynamic interactions that may change over time. The relationships between omics data collected at a single time point during a transition may not be informative. Hence, multi-omics experiments are further augmented with a time-course dimension to explore the temporal dynamic relationships between biomolecules [3].

While performing the integration of time-course multi-omics data, two aspects of the data should be considered. The first aspect is the difference in the expression level. The importance of this could be highlighted by the popularity of tools that detect differentially expressed sites, such as *DESeq2* [4], *limma* [5], and *edgeR* [6]. The second aspect is the temporal dynamic relations across distinct omics modalities [7, 8]. In particular, we will concentrate on within-gene correlation analysis, which is designed to study how much the change in one modality (*e.g.* proteiomics, methylation, etc.) of one gene across conditions can be explained by the change of the corresponding mRNA levels, as defined by [9]. This is in contrast to cross-gene analysis, which considers the correlation between mRNA abundance and the score of another modality across the different genes under a given condition. Consistent temporal patterns between time-courses from different modalities imply potential underlying regulatory relationships. For example, in transcriptomic-proteomic correlation, it is reported in [10] that only a limited average correlation between mRNA level and protein concentration (*ρ* = 0*.*41) is observed and most mRNA transcripts have no directly related temporal expression with the corresponding proteins. However, genes participating in core metabolic pathways present the highest within-gene correlation [9]. As for the mRNA level-DNA methylation level correlation, in non-CpG island promoters, there is a predominant trend of negative correlation between DNA methylation and mRNA levels. Meanwhile, in distal regulatory elements, both positive and negative correlations have been detected [11]. For the mRNA level-chromatin accessibility correlation, the mRNA abundance of transcription factors directly correlates with the accessibility of the corresponding binding sites, as demonstrated with examples from the *sHox* and *Gata* families [12]. Hence, we need to prioritize our focus on pairs that have a high correlation.

**Fig. 1:**
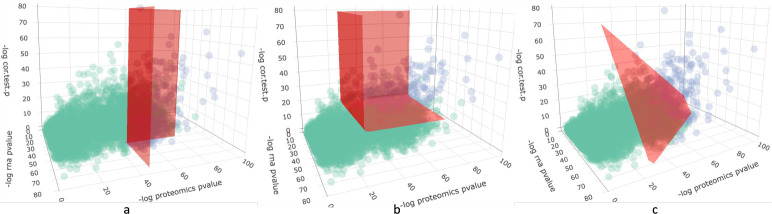
These plots illustrate how sites are selected using different methods. Each dot represents a gene. The *x*-axis and *y*-axis correspond to the −log*P*-value of mRNA and protein differential expression, respectively (see details in Section 2). The *z*-axis is the −log*P*-value of the correlation between mRNA level and protein level. The red plane separates the selected genes (blue) and the non-selected genes (green). a: In commonDE, the correlation is not considered. b: In MINT-DE with Edgington’s method, the separating plane has a curved shape in the log scale. But it is flat without log transformation, just like c. c: In MINT-DE with Fisher’s method, the separating plane is flat after log transformation.

Methods that integrate time-course multi-omics data are still in their infancy, and those fulfilling the pursuit of both aspects remain scarce. A naive approach is simply to select sites that are differentially expressed simultaneously in all assays as a first step to subset sites [13–16]. Such methods, referred to here as the “commonDE” method, only focus on the differential expression while neglecting the temporal similarity across modalities. This would cause one to overlook sites that are co-expressed across assays but have only moderate differential expression over time in one of the modalities (as shown in Figure 1a). Those sites might also be biologically informative and should be considered in downstream analysis, offering complementary information that would not be derived from analyzing one assay alone. Therefore, a method that selects sites based on the summary of information from all modalities is needed.

Another type of method that attracts considerable attention is based on factor analysis, such as *timeOmics* [17] and MEFISTO [13]. Factor analysis-based methods assume that there are a few latent factors that generate different omics. *timeOmics* can further perform site selection by sparse penalization. However, there are two drawbacks with methods based on sparse factor analysis such as *timeOmics*. First, they fail to explicitly incorporate the range of profile changes into the model. This will lead to the selection of sites with relatively lower differential expression as opposed to those with the largest differential expression. To alleviate this problem, an *ad hoc* method is proposed in [17], which is to preprocess data with filtering that removes genes with low coefficients of variation. Nonetheless, this sort of filtering is not sufficient to prioritize candidates with larger magnitudes of change. Second, these methods only provide a summary level description but lose granularity at the individual site level. By individual granularity, we mean sites with linearly correlated expression patterns may be equally important and should be included in the selection if differentially expressed. However, as site selection is achieved using sparse penalization like Lasso [18, 19] in *timeOmics*, only the most representative sites would be chosen. Therefore, they are not suitable for tasks where the information of individual time-courses is critical, such as identifying which sites (genes) are most important in a biological process.

We propose a straightforward framework, MINT-DE, which simultaneously considers the magnitude of change of each individual profile and the temporal concordance across assays, for site selection. In brief, MINT-DE first calculates *P*-values to test the significance of differential expression of a site in each assay. Then MINT-DE computes a *P*-value to test the correlation between time-courses of different assays, reflecting their temporal concordance. Then *P*-values are summarized as an overall measurement of the importance of site-pairs (see Figures 1b and 1c).

To evaluate the performance of MINT-DE, we applied it to analyze the *Drosophila* developmental dataset found in [10]. Quantitatively, we found the time-courses selected by our method had overall greater ranges of change compared to latent factor-based methods and the highest within-gene correlations compared to all other methods. Moreover, the gene-gene interaction network featured the highest degrees based on the gene set selected by MINT-DE, compared to the commonDE method and *timeOmics*. Qualitatively, in the Gene Ontology (GO) term analysis, the enriched GO terms based on the genes selected by MINT-DE provided a complementary interpretation of the genes involved in *Drosophila* embryogenesis.

## 2 Methods

Given multi-omics, we aim to find a shortlist of genes that are differentially expressed in at least one assay while also sharing similar temporal pattern across assays. We will start from two assays and assume one has log fold change (LFC) against the baseline, $\left\{x_{tg}\right\}$, for the first assay (i.e., gene expression level), where g=1,…,G, t=0,1,…,T,G is the number of sites sequenced, and T is the total number of time points. For the other assay (i.e., proteomics), one has $\left\{y_{tg}\right\}$, where g=1,…,G, and t=0,1,…,T. We assume that $x_{tg}=\mu_{tg}^{X}+\epsilon_{tg}^{X}$ and $y_{tg}=\mu_{tg}^{Y}+\epsilon_{tg}^{Y}$, where $\mu_{tg}^{X}=E\left(x_{tg}\right)$, $\mu_{tg}^{Y}=E\left(y_{tg}\right)$, and $\epsilon_{tg}^{X},\epsilon_{tg}^{Y}∼N\left(0,\sigma_{g}^{2}\right)$.

The $x_{tg}$ and $y_{tg}$ could also be count numbers with appropriate normalization, and from different number of biological replicates. Then we can concatenate the block of each biological replicate in a rowwise fashion. But for simplicity, we assume they are in LFC with the same number of replicates.

Before presenting our method MINT-DE, we provide a brief description of two commonly used methods in the literature.

### 2.1 commonDE method

A simple yet useful approach, which we will call the commonDE method, is to simply select sites with magnitudes of change of time-courses exceeding specified thresholds that are common in both assays [20]. To achieve this, one (i) calculates the *P*-value based on the null hypothesis that the site is not differentially expressed at each time point by using the LFC at baseline, $x_{0g}$, as control, and the LFC afterwards, $x_{tg}$, as treatment, with limma or DESeq2, for t=1,…,T and g=1,…,G. That is, for each site g, we can test the null

$$H_{0}\;:\mu_{1g}^{X}=\mu_{2g}^{X}=\cdots =\mu_{Tg}^{X}=0$$

against the alternative:

$$H_{A}\;:\;\text{at}\;\text{least}\;\text{one}\;\mu_{tg}^{X}\neq 0\;\text{for}\;t=1,\ldots ,T$$

This hypothesis testing could be achieved in two ways. The first way is to perform pairwise testing with the alternatives:

$$H_{At}\;:\mu_{0g}^{X}\neq \mu_{tg}^{X}\;\text{for}\;t=1,\ldots ,T\text{.}\;$$

The second approach is to use a spline model as provided in limma with voom transformation [21]. The *P*-value for gene g at time t is denoted as $p_{tg}^{X}$. Then one takes the minimum of P-values across all time points. Hence, (i) could be summarized with the expression, $p_{g}^{X}=min(p_{1g}^{X},p_{2g}^{X},\ldots ,p_{Tg}^{X})$. (ii) repeat (i) with $y_{tg}$ and get $p_{g}^{Y}$. Next, one picks up the intersection of sites with both $p_{g}^{X}$ and $p_{g}^{Y}$ below certain specified thresholds τ, that is, $G_{\tau }=\left\{g\;:p_{g}^{X}<\tau \right.\;and\;\left.p_{g}^{Y}<\tau \right\}$ (the threshold for different assays can vary). However, this commonDE method does not incorporate the information across assays, thus not capable of discovering inter-assay relations.

### 2.2 Factor analysis method

The other approach is to use a latent factor-based method, exemplified with *timeOmics* [17]. The *timeOmics* approach uses sparse partial least square (sPLS) [18] to find H latent factors for given matrices $X,Y\in R^{T\times G}$ separately, such that the correlation coefficients between latent factors are maximized with sparse penalty. The loadings of genes onto each latent factor, denoted as $\left(u_{1},\ldots ,u_{H}\right)$ for X and $\left(v_{1},\ldots ,v_{H}\right)$ for Y, can be sparsely determined, such that each latent factor has a specified number, say $l_{h}$, of loadings for h=1,…,H. The optimization problem is:

$$\underset{u,v}{min}\;{∥X_{h}^{T}Y_{h}-uv^{T}∥}_{F}^{2}+P_{\lambda_{1}}(u)+P_{\lambda_{2}}(v),$$

where $X_{h}$ and $Y_{h}$ are deflated data at the hth step and $P_{\lambda }(u)$ is a penalty function. Then the latent factors could be computed by $\xi_{h}=X_{h}u_{h}$ and $\omega_{h}=Y_{h}v_{h}$. The total $\sum_{h=1}^{H}\;l_{h}$ loading genes could be interpreted as the selected genes.

In the latent factor-based method, the magnitudes of change are not explicitly modeled in the formulation. Consequently, weak signals or noise may be selected, while differentially expressed sites are neglected. Even though an *ad hoc* low count removal is suggested to be used in preprocessing [17], those profiles with relatively lower differential expression would still have an equal chance to enter the selection compared with those differentially expressed ones. Another issue of using a summary set of time-courses is the loss of individual site granularity. Due to the penalty that enforces sparsity, only a representative subset of linearly dependent sites would be selected, which leads to the possibility of dropping important sites that are colinear to the selected ones. It is possible that sites sharing a similar expression pattern might be involved in related biological processes or in the same pathways.

### 2.3 Our Method: MINT-DE

To overcome the issues in the *timeOmics* and the commonDE approaches, we propose MINT-DE that simultaneously considers the significance of the expression change and the temporal concordance across assays. Based on (i)-(ii) mentioned in Section 2.1, we further (iii) compute the *P*-values of the correlation test (Spearman or Peason correlation test) between $x_{.g}$ and $y_{.g}$, denoted as $p_{g}^{XY}$ for gene g. Then we (iv) use either the Edgington method [22]

$$S_{g}=p_{g}^{X}+p_{g}^{Y}+p_{g}^{XY}$$

or Fisher’s method [23]

$$S_{g}^{\prime }=2\log\left(p_{g}^{X}\right)+2\log\left(p_{g}^{Y}\right)+2\;log\left(p_{g}^{XY}\right)$$

to summarize the *P*-values. Then sites with the smallest summarized *P*-values can be identified. Such a summarized statistic takes both significance of LFC difference ($p_{g}^{X}$ and $p_{g}^{Y}$ from limma) and significance of temporal consistency ($p_{g}^{XY}$ from correlation test) into consideration, unlike the commonDE which only incorporates the magnitude of change or the latent factor-based methods which only consider temporal consistency.

A general discussion of desiderata for combining evidence measures is given in [24], and it is shown that Fisher and (a method equivalent to) Edgington approaches both have coherent properties. However, Fisher’s method can be overwhelmed by small *P*-values, while Edgington’s method is sensitive to large *P*-values [25]. This is an exploitable property of Edgington’s method in practice since if any of the three *P*-values is too large, which implies that a gene has either a flat expression in an assay or a low within-gene correlation, then it will not be selected. Fisher’s method is optimal for an alternative where *P*-values follow a beta distribution (Beta(a,1), for 0<a<1), whereas the Edgington method is optimal for an alternative where *P*-values have a truncated exponential distribution (${Exp}_{\left[0,1\right]}(b)$, for b>1) [26]. As in Figure 1, the selection plane separating significant sites from non-significant sites is rendered flexible in three dimensions so that their temporal consistency becomes an additional dimension of consideration, and the site having a high correlation but a moderate signal in one of the assay now has the chance of entering the selection.

## 3 Results

To illustrate the potential of MINT-DE as an effective way to select sites differentially expressed within at least one assay and temporally correlated across assays, we analyzed a dataset on the development of fruit flies taken from [10] with MINT-DE. This dataset consists of paired transcriptome and proteome time-courses with 14 time points during *Drosophila* embryogenesis. These measurements were taken every hour for the first 6 hours after egg-laying, and every 2 hours afterwards until 20 hours, from four biological replicates. mRNA levels were normalized and converted to LFC via a *voom* transformation. In the context of time-course of mRNA-level and protein-level analysis, the unit of inference is genes, so in this section we would adhere to this label rather than the previous term “sites”.

We selected 200 genes using the different methods described in Section 2 (our results were qualitatively similar when we replicated with top 100 genes, see Figure A2). In the commonDE method, this was achieved by setting the threshold, τ of both *P*-values obtained from *limma*, as described in Section 2.1, to be $\tau =10^{-11.6}$. In MINT-DE, this was accomplished by ordering the sum of the *P*-values (Edgington’s method) and selecting the 200 genes with the smallest sums (MINT-DE using Fisher’s method is also given in Figure A2). In *timeOmics*, this was obtained by setting the number of latent factors to H=4 with $l_{1}=l_{2}=l_{3}=l_{4}=50$ loadings in each factor.

Both quantitative and qualitative evaluations suggest that MINT-DE can select biologically meaningful candidates missed by other methods. First, in Section 3.1, we will showcase that MINT-DE selects differentially expressed genes with high within-gene correlations by comparing with other methods. Next, in Section 3.2, we create two synthetic assays using the biological replicates, and assess which methods can pick up interesting genes. After that, in Section 3.3, we compare interactions among selected genes using the STRING database [27]. Finally, in Section 3.4, we conduct a GO enrichment analysis of the selected genes.

### 3.1 Contrasting gene selection methods

**Fig. 2:**
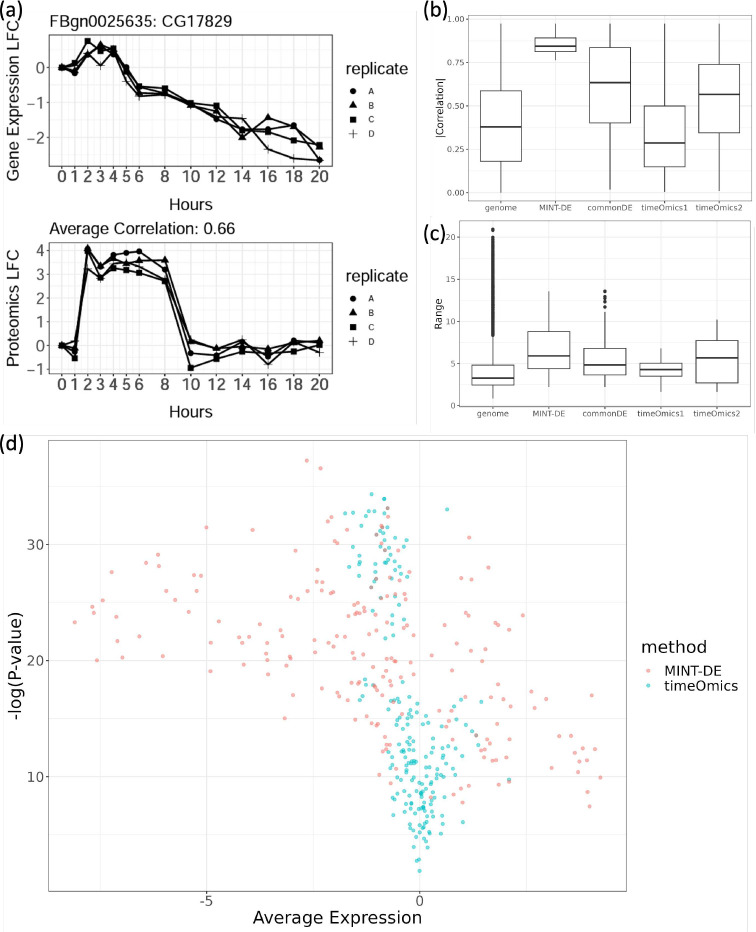
a: The transcripomic (upper) and proteomic (lower) time-courses of gene CG17829, with only moderate correlation across four replicates. b: Absolute value of within-gene correlations between mRNA level time-course and protein level time-course based on different methods. “genome” corresponds to the within-gene correlation distribution of all genes. “timeOmics1” and “timeOmics2” corresponds to the selection based on loading *u* and *v* respectively (see Section 2.2). c: Range of time-courses of selected genes by different methods. d: *P*-values of a spline model fitted for mRNA time-course vs. average LFC. The *timeOmics*’ selections have smaller average expression compared with those by MINT-DE.

To demonstrate that the commonDE method might select genes with widely different trajectories across the two assays, we first plotted the gene expression level and the corresponding protein level trajectories in Figure 2a. We observed that the commonDE method selected a gene with quite dissimilar dynamic patterns. This came with no surprise, since the commonDE method did not take correlation across modalities into consideration. Such dynamical discrepancies were generally observed in other selected time-course pairs, as exemplified by trajectories of 6 randomly sampled genes shown in Figure A1b. On the contrary, MINT-DE explicitly incorporated the correlation between the pair of time-courses, and overall temporal consistencies were widely observed. As a consequence, the within-gene correlations, shown in Figure 2b, between the selected pair of time-courses by MINT-DE were significantly higher than those selected by commonDE.

To probe if the *timeOmics* method might select genes with lower LFC in spite of good temporal concordance across the two assays, the ranges of the selected genes, defined as

$$\mathrm{range}\left(x_{g}\right)=\underset{t,b}{max}\;x_{tg}^{\left(b\right)}-\underset{t,b}{min}\;x_{tg}^{\left(b\right)},$$

were calculated and shown in Figure 2c. The distributions of ranges demonstrate that the pairs selected by *timeOmics* do not have large enough magnitude of change compared with methods incorporating LFC (MINT-DE and commonDE). The ranges of selections by both the MINT-DE and the commonDE method are overall larger than those by *timeOmics*, while, on the contrary, the LFC distribution of *timeOmics*’s selection is similar to a randomly chosen set of genes from the genome. This implies that without explicitly incorporating expression level into analysis, methods based on latent factor like *timeOmics* are prone to flagging genes with trivially small changes. But MINT-DE could select relatively differentially expressed genes.

To further illustrate how different the gene selections were, we first fitted the gene expression level time-courses with a spline of order 5 with *limma* and plotted −log_10_(*P*-value) vs. average expression for the site over all time and replicates (Figure 2d). The *x*-axis is the average of the gene expression in LFC, which approximates the extent of change throughout time, while the *y*-axis represents how similar the replicates are. According to Figure 2d, we observe that the gene selections based on *timeOmics* are concentrated around the *y*-axis, since it can not specifically choose those genes with large LFC. On the contrary, MINT-DE tends to choose genes that are more scattered away from the *y*-axis with some overlap with the commonDE method’s selection (see Figure A3) since both method detect those genes having large LFC.

### 3.2 Synthetic multi-omics data

When comparing the performance of different integration methods on real omics data, it is hard to know the truly correlated time-courses. So we compared different methods by constructing “pseudo-assays”. In particular, time-courses of biological replicates 1 and 2 were treated as one type of assay, and replicates 3 and 4 were regarded as the second type of assay. We expect a reasonable method to select genes with low variability across two pseudo-assays, since they are indeed biological replicates with similar expression levels throughout the time, therefore, genes with low variability across replicates should be prioritized in selection.

The variability was quantified by the coefficient of variation (CV) of time-courses of each gene across all biological replicates in each assay averaged over all time points, *i.e.*

$$cv\left(x_{g}\right)=\frac{1}{T}\sum_{t=1}^{T}\;\;\frac{sd\left(x_{tg}\right)}{mean\left(x_{tg}\right)},$$

$$sd\left(x_{tg}\right)=\sqrt{\frac{1}{B}\sum_{b=1}^{B}\;\;{\left(x_{tg}^{\left(b\right)}-mean\left(x_{tg}\right)\right)}^{2},}$$

$$\mathrm{mean}\left(x_{tg}\right)=\frac{1}{B}\sum_{b=1}^{B}\;\;x_{tg}^{\left(b\right)}.$$

**Fig. 3:**
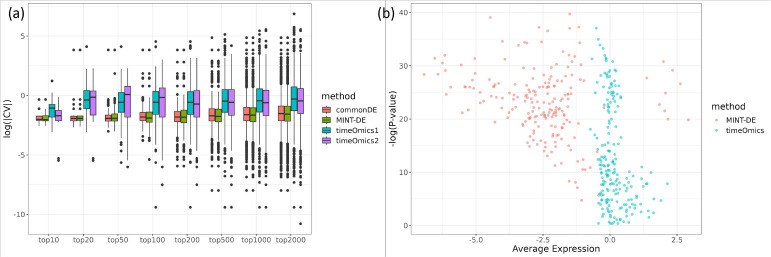
a: CV of gene expression levels of different-sized collections of genes selected by the different methods, including commonDE, MINT-DE, timeOmics1 (the *timeOmics* selection based on biological replicates 1 and 2) and timeOmics2 (based on *timeOmics* selection from replicates 3 and 4). Both timeomics samples have larger CV compared to other methods, implying that there is greater variability between the selected time-course replicates. b: *P*-values vs. average LFC of a spline model fitted for mRNA time-course with *limma*. The *timeOmics*’ selections have smaller average expression compared with those by MINT-DE.

To assess the robustness of our finding, we compared the variability of the top n genes obtained by the MINT-DE, commonDE and *timeOmics* methods and reported the results for different values of n=10,20,50,100,200,500,1000 and 2000 (the parameters used in each method are summarized in Section A.2). Figure 3a shows that *timeOmics* yields an overall larger CV in both pseudo-assays of transcriptomics and proteomics, since it is designed for across-gene correlation but not for within-gene correlation.

*timeOmics* tends to identify genes with the most representative expression trajectory, however, this trajectory might be relatively flat across time (with lower LFC). To further illustrate this, we constructed a spline model for the selected mRNA time-course, and we observed a smaller average LFC (Figure 3b). This again supports the finding in the real data setting in Section 3.1 that the *timeOmics* selection has the smallest range (Figure 2b) and a relatively smaller average log LFC (Figure 2c).

### 3.3 Inferred gene-gene interaction networks

After comparing the performance of different methods on synthetic multi-omics data, we wanted to investigate whether the selection demonstrated evidence for a statistical enrichment of any known gene-gene connection or biological function in the literature. First, we assessed which methods provided genes involved in known gene-gene and protein-protein interaction networks.

For evaluation based on gene-gene interaction, after obtaining a short list of genes, we constructed a gene-gene interaction network using the STRING database [27]. The STRING database integrates gene-gene and protein-protein interactions into a single network with edges scored based on combined evidence from seven source channels [28], including *neighborhood*, *fusion* and *co-occurrence* for association gleaned from genome sequence, *co-expression* for functional genomics measurements indicating common expression regulation, *experiments* for laboratory experiments, *database* for protein–protein associations that are already known from textbook knowledge, and *textmining* for knowledge from article parsing. While constructing the network, all of seven sources were included. All other parameters were set in their default values.

In the STRING network, the connection between genes means either a direct physical binding or an indirect interaction, which could be involvement in the same metabolic pathway or cellular process [28]. Finding more densely connected nodes with a higher average node degree and higher clustering coefficients implies that there is more evidence supporting the idea that those genes are cooperating in the same metabolic pathway or cellular process and sharing similar biological functions. The density of node connections reflects the number of interactions that a gene/protein has on average, while the node degree reflects a measure of how connected the nodes are.

According to Table 1, the network constructed based on the genes identified by MINT-DE has the highest average degree (also the highest number of edges), compared with the other methods. This suggests that those genes selected by MINT-DE are actively interacting with each other and participating the same metabolic pathways or cellular process during fly embryogenesis. Therefore, they should be prioritized in the selection. The construction of such a denser gene-gene interaction network based on selection by MINT-DE is surprising, since the selection is based on within-gene correlation across modalities, but it also contains genes actively interacting with each other. In other words, genes undergoing synchronized alterations in transcription and translation levels might be together actively participating in fly embryogenesis. The network constructed with genes selected by *timeOmics*, is sparser than the one based on selection from MINT-DE, even though *timeOmics* selected profiles are highly correlated across genes. This further supports the importance of within-gene correlation across assays in multi-omics integration. It is also worth noting that, from the comparison between *timeOmics* with and without filtering, correlation alone is insufficient to detect gene sets with high interactions and the magnitude of differential expression must also be considered.

### 3.4 Validation by GO terms

In order to determine whether MINT-DE selected a set of biologically informative genes complementary to the selection criteria of other methods, biological process GO term enrichment analyses were performed to investigate whether certain biological functions were specifically enriched by the genes selected by MINT-DE. GO term analysis was executed by setting the selected gene list as “differentially expressed genes” and the remaining genes as “background” in the *clusterProfiler* package [29, 30].

**Fig. 4:**
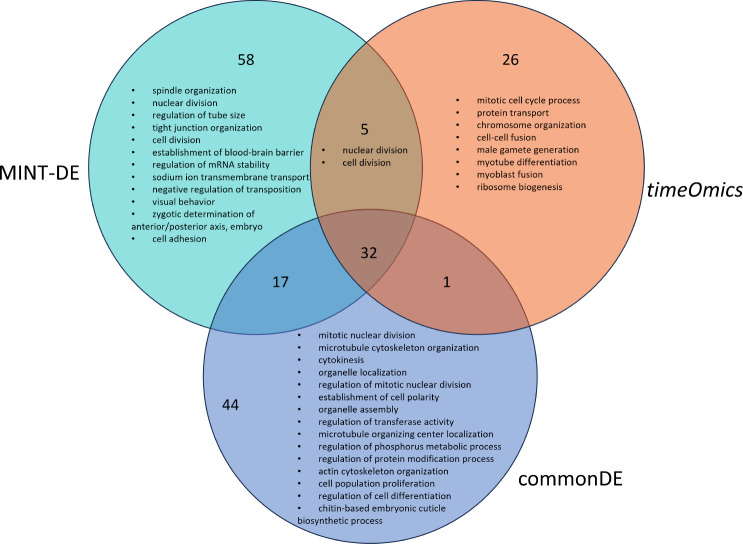
GO terms enriched based on genes selected by MINT-DE, *timeOmics*, and commonDE. The number represents the total number of significant GO terms. The text represents semantic summary of GO terms by *rrvgo*.

For MINT-DE, we found a total of 94 significant terms (adjusted *P*-values *<* 0*.*05), while *timeOmics* and the commonDE method yielded, respectively, 64 and 112 significantly enriched terms (see the Supplementary Material for full list of significant GO terms). Figure 4 shows the summary of the enriched GO terms clustered based on semantic similarity measures using *rrvgo* [31].

MINT-DE identifies genes essential for embryogenesis, successfully focusing on key processes including:

- Spindle organization: MINT-DE found genes including *Ncd*, *Klp3A*, and *Klp67A* crucial for building and maintaining the mitotic spindles needed for rapid cell division in the syncytial embryo [32].
- Blood-brain barrier formation: *Nrg* is a gene identified by MINT-DE involved in this late-embryonic process [33] (see Figure A1a).
- Tight junction organization: Septate junctions formed by subperineurial glia cells restrict paracellular diffusion and protect the nervous system, with MINT-DE identifying genes active during their mid- to late-embryonic development [33].
- Heart tube size regulation: Heart development starts at stage 10 of embryogenesis, and MINT-DE identified genes influencing tube size [34].
- mRNA stability: MINT-DE associated genes like *bol* to increased RNA stability and protein levels [35], while genes like *smg* (Figure A1a) were linked to RNA degradation and suppressed protein production.
- Transposon expression control: MINT-DE identified *Aubergine*, a gene involved in the PIWI pathway that regulates transposons in germ cells to maintain genome stability by suppressing transposon movement. Prior to zygotic genome activation, the maternally inherited PIWI protein is imported into the nuclei of somatic cells where it appears to be vital to the process of early embryo development [36].
- Sodium ion transport: MINT identified genes linked to this process, with altered expression harming development [37].

According to the Venn diagram of significantly enriched GO terms (Figure 4), MINT-DE enables researchers to find candidates of genes missed by methods concentrating on LFC or correlation only, since there are 58 specifically enriched GO terms for fly embryogenesis which are not identified by either commonDE or *timeOmics* alone. This implies that including both correlation and differential expression adds an important perspective to the relevance of genes.

## 4 Conclusions

Based on Edgington’s method of combining *P*-values from multiple hypothesis tests, we propose MINT-DE to select genes whose time-course multi-omics data have a high within-gene correlation across modalities and a differential expression in at least one modality. The case study in fly embryogenesis suggests the potential of this method to detect biologically meaningful genes missed by other methods. MINT-DE can serve as a first step towards integrative analysis of time-course multi-omics data.

Our study acknowledges certain limitations. Edgington’s method is based on the assumption that the *P*-values are obtained from independent tests, which has not been verified in our study. Since the aim of our study is to identify a subset of promising site candidates rather than rigorously controlling false discovery, the theoretical guarantee of false positive will addressed in future work.

There are several extensions of MINT-DE that could be easily achieved. Even though the case study is based on time-courses in transcriptomics and proteomics, MINT-DE could be easily extended to other types of multi-omics data, such as DNA methylation and ATAC-seq. MINT-DE is also not restricted to time-course data, it can be generalized to cross-sectional multi-omics data. In this case, the $p_{g}^{X}$ and $p_{g}^{Y}$ would be replaced with the *P*-values for differential expression of gene g between two conditions in two assays, and $p_{g}^{XY}$ would be replaced with the correlation across samples. Our method does not use correlation across time points (lagged correlation) due to coarse time resolution of data. However, the framework can be generalized by introducing *P*-values of partial autocorrelations across genes.

Besides the development of the method, this study also highlights the importance of considering both differential expression and within-gene correlation in time-course multi-omics study. For example, [9] finds that among genes whose protein products react swiftly to changes in their mRNA abundance, within-gene correlations tend to be the highest. Therefore, they suggest that analyses integrating transcriptomic and proteomic data should be conducted more routinely through either correlative analyses or mathematical modeling.

## Figures and Tables

**Table 1:** PPI Network statistics.

Method	# nodes	# edges	avg. node degree	avg. local clustering coef.	PPI enrichment *P*-value

MINT-DE	200	502	5.02	0.424	< 1.0e – 16
commonDE	195	445	4.56	0.447	< 1.0e – 16
*time Omics*	201	454	4.52	0.423	< 1.0e – 16
*timeOmics* ^†^	201	190	1.89	0.268	1.76e – 8

*timeOmics*^†^ is *timeOmics* without pre-filtering of low CV genes. The selection based on MINT-DE features a densest network.

## Data Availability

The code is available in https://github.com/xvehao/TimeCourseIntegromics.

## Abbreviations

GO: Gene Ontology
LFC: Log fold change
sPLS: Sparse Partial Least Square
CV: Coefficient of variation
