# Supplementary material for: Integrative Analysis of Differentially Expressed Genes in Time-Course Multi-Omics Data with MINT-DE
Source: Res Sq. 2024 Dec 23:rs.3.rs-3806701. Originally published 2024 Jan 1. Preprint. [Version 2] doi: 10.21203/rs.3.rs-3806701/v2 (PMC10802680; doi:10.21203/rs.3.rs-3806701/v2)
Supplement: Supplement 1 [file NIHPPRS3806701V2-supplement-1.pdf]

Supplementary Files

This is a list of supplementary files associated with this preprint. Click to download.

- suppmatGO.xlsx
- Appendix.pdf
- FigA1a.png
- FigA1b.png
- FigA2.png
- FigA3.png
- FigA4.png
- FigA5.png
